# Production of proinflammatory mediators in activated microglia is synergistically regulated by Notch-1, glycogen synthase kinase (GSK-3β) and NF-κB/p65 signalling

**DOI:** 10.1371/journal.pone.0186764

**Published:** 2017-10-19

**Authors:** Qiong Cao, Aparna Karthikeyan, S. Thameem Dheen, Charanjit Kaur, Eng-Ang Ling

**Affiliations:** Department of Anatomy, Yong Loo Lin School of Medicine, National University of Singapore, Singapore; Lewis Katz School of Medicine at Temple University, UNITED STATES

## Abstract

Microglia activation and associated inflammatory response are involved in the pathogenesis of different neurodegenerative diseases. We have reported that Notch-1 and NF-κB/p65 signalling pathways operate in synergy in regulating the production of proinflammatory mediators in activated microglia. In the latter, there is also evidence by others that glycogen synthase kinase 3β (GSK-3β) mediates the release of proinflammatory cytokines but the interrelationships between the three signalling pathways have not been fully clarified. This is an important issue as activated microglia are potential therapeutic target for amelioration of microglia mediated neuroinflammation. Here we show that blocking of Notch-1 with *N*-[(3,5-Difluorophenyl) acetyl]-L-alanyl-2-phenylglycine-1,1-dimethylethyl ester (DAPT) in LPS activated BV-2 microglia not only suppressed Notch intracellular domain (NICD) and Hes-1 protein expression, but also that of GSK-3β. Conversely, blocking of the latter with lithium chloride (LiCl) decreased NICD expression in a dose-dependent manner; moreover, Hes-1 immunofluorescence was attenuated. Along with this, the protein expression level of p-GSK-3β and p-AKT protein expression was significantly increased. Furthermore, DAPT and LiCl decreased production of IL-1β, TNF-α, IL-6, iNOS, Cox2 and MCP-1; however, IL-10 expression was increased notably in LiCl treated cells. The effects of DAPT and LiCl on changes of the above-mentioned biomarkers were confirmed by immunofluorescence in both BV-2 and primary microglia. Additionally, NF-κB/p65 immunofluorescence was attenuated by DAPT and LiCl; as opposed to this, IκBα protein expression was increased. Taken together, it is suggested that Notch-1, NF-κB/p65 and GSK-3β operate in synergy to inhibit microglia activation. This may be effected via increased expression of phospho-GSK-3β (p-GSK-3β), phospho-protein kinase B (PKB) (p-AKT) and IκBα. It is concluded that the three signalling pathways are functionally interlinked in regulating microglia activation.

## Introduction

Microglia are immunocompetent cells in the central nervous system whose activation is implicated in different neurodegenerative diseases or neurological disorders (see Dheen et al. [1]). It is well documented that microglia activation is regulated by different signaling pathways involved in production of proinflammatory mediators such as TNF-α, IL-1β, nitric oxide and ROS which are deleterious to neurons and oligodendrocytes [1]. Among the various signaling pathways that regulate microglial activation include MAPK p38 [2], Nuclear Factor- κB /p65 (NF-κB /p65) [3], glycogen synthase kinase (GSK-3β) [4–6] and Notch-1 [7–8]. In view of this, the various pathways have been considered to be potential therapeutic targets for amelioration of neuroinflammation triggered by activated microglia causing neuronal or oligodendrocyte death [9].

In our previous studies, we have reported that microglia constitutively express Notch-1 and NF-κB /p65 [3,7]; both pathways play important roles in cellular functions under physiological and pathological conditions. Furthermore, we have demonstrated the functional roles of Notch-1 and NF-κB /p65 in activated microglia *in vivo* and *in vitro* [3,7,10–11]. More importantly, we have shown for the first time the interrelation between Notch-1 and NF-κB /p65, emphasizing that both pathways synergistically modulate the proinflammatory function in activated microglia. More specifically, Notch signaling can amplify the proinflammatory response of microglia by enhancing the NF-κB /p65 signaling. It remained to be further determined how the two pathways might work in concert to regulate the microglial cell functions.

Glycogen synthase kinase GSK-3β is a multifunctional kinase whose activity is regulated by serine (inhibitory) and tyrosine (stimulatory) phosphorylation [12]. GSK-3β expression is ubiquitous in eukaryotes and plays multiple roles in various essential physiological and pathological processes including glycogen metabolism, cell cycle control, apoptosis, embryonic development, cell differentiation, cell motility, microtubule function, cell adhesion and inflammation [13–15]. It is part of the molecular machinery regulating the adaptive response to lipopolysaccharide (LPS) stimulation in microglial cells [4]. Furthermore, it regulates toll-like receptor [16] and controls the production of interleukin-6 after LPS engages with toll-like receptors [4]. GSK-3β mediates the release of interleukin-1β, TNF-α and IL-10 from cortical glia [17].

It has been reported that phosphorylation of GSK-3β down-regulates Notch-1 activity [18]. Both GSK-3β and Notch-1 can modulate production of proinflammatory cytokines in activated microglial cells [19–20]. In consideration of previous study demonstrating the functional relationship between Notch and NF-κB in modulating microglial activation, we speculate that GSK-3β may act as a link between Notch and NF-κB in the modulation of proinflammatory function in microglial cells. In light of this, we sought to determine the functional relationship between GSK-3β, Notch-1 and NF-κB/p65 in activated microglia. We used lithium chloride (LiCl) that is known to directly inhibit GSK-3β through binding to the magnesium-sensitive site of the enzyme [21–22], and indirectly inhibit it by enhancing phosphorylation of specific serine residues. The phosphorylation of GSK-3β by activating upstream AKT and protein kinases A or C can then result in different effects on various processes [23–24]. N-[N-(3,5-Difluorophenacetyl)-L-alanyl]-S-phenylglycine t-butyl ester (DAPT) was used as an inhibitor for Notch via γ-secretase complex in microglial cells challenged with LPS. The treated and untreated microglia were then subjected to real time polymerase chain reaction (RT-PCR) analysis, immunofluorescence labeling, Western blotting and enzyme-linked immunosorbent assay (ELISA).

## Materials and methods

### Ethics statement

All experiments were carried out in accordance with the National Institutes of Health Guide for the care and use of laboratory animals (NIH publications No. 80–23). Use of animals was approved from National University of Singapore (NUS) Institutional Animal Care and Use Committee [**IACUC No.** R13-5512(A)15]. This study was approved by NUS, Project No. R-181-000-140-592 (E-A L). The animals were housed at room temperature (25°C) and 12h of light and dark cycle. All efforts were made to reduce the number of rats used for primary microglial culture.

### Expression of GSK-3β, Notch-1 and proinflammatory mediators in BV-2 cells treated with different doses of LiCl and DAPT and with LPS

Murine BV-2 cells were cultured in Dulbecco’s modified Eagle’s medium (DMEM) supplemented with 2% FBS incubated in a humidified incubator with 5% CO_2_, 95% air at 37°C. The confluent BV-2 cells were plated in a six-well plate at a density of 1.5X10^6^ incubated under different conditions at 37°C in a humidified incubator with 5% CO_2_/95% air for 24h.

For determination of GSK-3β expression after LiCl treatment, BV-2 cells were divided into five groups. Group 1, BV-2 cells were untreated; group 2, BV-2 cells were stimulated with LPS for 4h; group 3–5, BV-2 cells were treated with LiCl at 15mM, 20 mM and 25mM, respectively, for 0.5h and then challenged with LPS for 4h. In a separate group, BV-2 cells were divided into six groups comprising untreated, LPS and DAPT at 10 μM, 15μM, 20 μM and 25μM pretreatment +LPS to determine the effects on Notch-1 and GSK-3β expression. In both untreated and untreated BV-2 cells, RNA and proteins were harvested for RT-PCR and Western blot analysis, respectively.

### BV-2 cells treated with DAPT at 20μM and LiCl at 20mM were selected from the above to investigate their response to changes of Notch pathway, GSK-3β and its upstream AKT

These dosages were selected based the observations that NICD expression was drastically suppressed by LiCl at 20mM, and DAPT at 20μM. In this regard, expression of NF- κB/p65 and IκB a as well as the functional response such as production of proinflammatory cytokines IL-1β, IL-6 and TNF-α, stress mediator iNOS and COX2, anti-inflammatory cytokine IL-10, and the chemokine MCP-1 involved in microglial migration was analyzed. For this, four groups of BV-2 cells were subjected to different treatments. In group 1, the cells were incubated in DMEM with 2% FBS without treatment. In group 2, the cells were treated with LPS for 4 h. In group 3, the cells were treated with DAPT at 20μM (Sigma-Aldrich, MO, USA) for 3 h and then challenged with LPS for 4h. In group 4, the cells were treated with LiCl at 20mM for 0.5h and then challenged with LPS for 4h. Some cell samples were fixed in 4% paraformaldehyde in 0.1 M phosphate buffer (PB) for 15 min and processed for immunofluorescence labeling; others were used for RT-PCR and Western blotting. All experiments were carried out at least in triplicate.

### Immunofluorescence labeling

BV-2 cells plated on coverslips coated with poly-L-lysine at a density of 1x10^6^ cells/ml cells per well were treated with LPS, DAPT+LPS and LiCl+LPS, respectively. The cells were fixed in 4% paraformaldehyde in 0.1 M PB for 15 min. After rinsing with PBS, the coverslips with adherent cells were used for double immunofluorescence labeling. BV-2 cells were incubated in goat anti-rabbit activated Notch-1(NICD) polyclonal antibody (dilution 1:500; Abcam, incorporated in the UK), goat polyclonal antibody Hes-1 (dilution 1:200; Santa Cruz Biotechnology), goat anti-rabbit GSK3β (dilution 1:100; Santa Cruz Biotechnology), goat anti-rabbit p-GSK 3β(ser9) (1:250; Cell Signaling Technology), goat anti-rabbit p-AKT(ser473)(1:250; Cell Signaling Technology), goat anti-rabbit IL-1β (dilution 1:500; Chemicon, Temecula, CA), anti-mouse TNF-α antibody (dilution 1:500; Chemicon), anti-mouse IL-10 antibody (dilution 1:200; Santa Cruz Biotechnology) and goat-anti-rabbit MCP-1(1:200; Santa Cruz Biotechnology)(Table 1) Subsequently, the cells were incubated with FITC-conjugated and Cy3-conjugated secondary antibodies for 1 h at room temperature. The coverslips were mounted on the slides with histology Mounti-Fluoroshield with DAPI (Sigma, CA, USA). All images were captured with a confocal microscope (Fluoview1000; Olympus, Tokyo, Japan).

**Table 1:** List of antibodies for immunofluorescence.

Name of antibody	source	dilution	Company Name	Catalogue number
NICD	rabbit	1:250	Abcam Singapore	Ab8925
Hes-1	mouse	1:100	Santa Cruz Biotechnology. INC	SC166410
GSK-3β	rabbit	1:100	Santa Cruz Biotechnology. INC	SC-9166
p-GSK3β(Ser9)	rabbit	1:250	Cell Signalling technology	9336
p-AKT (Ser 473)	rabbit	1:250	Cell Signalling technology	9271
NFκB	mouse	1:250	Cell Signalling technology	6956
TNF-α	rabbit	1:200	Chemicon	1837
IL-1β	rabbit	1:200	Chemicon	1832P
IL-10	mouse	1:200	Santa Cruz Biotechnology. INC	SC-365858
MCP-1	rabbit	1:100	Santa Cruz Biotechnology. INC	SC-28879
CD11b	mouse	1:300	BD transduction Laboratories	610442

### RT-PCR analysis

Total RNA was extracted from BV-2 cells of all groups using the RNeasy Mini kit (Qiagen, Valencia, CA). Reverse transcription reactions were performed using the reverse transcription system kit (catalog No. 3500; Promega, Singapore). The resultant cDNA was diluted 10 times in double-distilled H_2_0 and kept at -20°C for RT-PCR analysis. Gene sequences for primer design were obtained from the National Centre for Biotechnology Information’s Gene Bank. Primer pairs for GSK-3β, IL- 1β, IL- 6, TNF-α, iNOS and β-actin were designed using the primer design program (Primer 3 software version 1.0). Forward and reverse primer sequences for the genes and their corresponding amplicon size are listed in Table 2. RT-PCR was performed using a 96-well plate run in the ABI machine according to the manufacturer’s instructions. The RT-PCR was briefly carried out in a 10μl final volume containing the following: SYBR Green I master mix (Qiagen); 1μl of 5μM forward primer and reverse primer, respectively, and 3μl of diluted cDNA. After an initial denaturation step at 95°C for 15 min, temperature cycling was initiated. Each cycle consisted of denaturation at 94°C for 15 sec, annealing at 60°C for 25 sec, and elongation at 72°C for 20 sec. In total, 45 cycles were performed. Mouse β-actin was amplified as the control for normalizing the quantities of transcripts of each of the above-mentioned genes. The expression differences for GSK-3β, IL-1β, IL-6, TNF-α and iNOS between the control and treated cells were calculated by normalizing with the β-actin gene expression according to the following formula [25]: Fold change 5 22[Ct (control) gene X–Ct (control) actin]–[Ct (activated) gene X–Ct (activated) actin].

**Table 2:** List of primers for RT-PCR.

Name of primer	Sequence		Size
	left	right	
GSK-3β (Ms)	ccccacacatctgctaaggt	tacacacacggtcggagaag	200bp
IL-1β (Ms)	gcccatcctctgtgactcat	aggccacaggtattttgtcg	229bp
IL-6 (Ms)	cttccatccagttgccttct	tccacgatttcccagagaac	168bp
TNF-α(Ms)	cgtcagccgatttgctatct	cggactccgcaaagtctaag	205bp
iNOS(Ms)	ctcactgggacagcacagaa	gcttgtctctgggtcctctg	217bp
β-actin(Ms)	agccatgtacgtagccatcc	gctgtggtggtgaagctgta	222bp

### Western blotting analysis and ELISA

The cell supernatant from different treated groups was collected and kept it in a -80°C freezer for ELISA. Culture medium was removed from the culture plate, and BV-2 cells were washed twice with ice-cold PBS. Cells were lysed with lysis buffer, mechanically scraped off with a rubber police- man, and centrifuged at 13,000 rpm for 25 min. Protein concentration of samples was then determined by using a protein assay kit (Bio-Rad, Hercules, CA; catalog No. 500–0002). Next, the protein samples were separated on 4–20% pre-casted sodium dodecyl sulfate-polyacrylamide gels. The proteins embedded in the gel were then transferred to the immobile polyvinylidene difluoride transfer membranes using a semidry electrophoretic transfer cell (Bio-Rad, Hercules, CA). The membranes were washed with TBS+0.1% Tween buffer and then incubated with 5% nonfat dry skim milk for 1h at room temperature. After this, they were incubated with goat anti-rabbit activated Notch-1(NICD) polyclonal antibody (dilution 1:1000; Abcam, incorporated in the UK), goat anti-rabbit deltex dilution 1:1000; Santa Cruz Biotechnology), goat anti-rabbit GSK-3β (dilution 1:1000; Santa Cruz Biotechnology), goat anti-rabbit phospho-GSK-3β (Ser9) (dilution 1:1000; Cell Signaling Technology), goat anti-rabbit phospho-AKT (Ser473) (dilution 1:1000; Cell Signaling Technology), anti-mouse NF-κB /p65 ((dilution 1:1000; Cell Signaling Technology), anti-mouse IκBα (dilution 1:1000; Cell Signaling Technology), anti-mouse iNOS (dilution 1:1000; BD), anti-mouse TNF-α (dilution 1:1000; Sigma-Aldrich), anti-mouse Cox2 (dilution 1:2000; BD), goat anti-rabbit MCP-1(dilution 1:1000; Santa Cruz Biotechnology), anti-rabbit IL-1β (dilution 1:1000; Chemicon) and anti-mouse β-actin (dilution 1:10000; Sigma-Aldrich) (Table 3) overnight on a shaker in a cool room. After three washes with TBS-0.1% Tween, the membranes were incubated with horseradish peroxidase-conjugated secondary antibody (dilution 1:5000; Pierce Biotechnology, Rockford, IL) for 1h. The above-mentioned proteins were detected with a chemiluminescence detection system according to the manufacturer’s instructions (Supersignal WestPico Horseradish Peroxidase Detection Kit; Pierce Biotechnology) and developed on the film. The band intensity of these protein expression levels relative to the β-actin was quantified in Image J software (NIH). All experiments were repeated at least in triplicate.

**Table 3:** List of antibodies for Western blotting.

Name of antibody	source	dilution	Company Name	Catalogue number
Activated Notch-1 (NICD)	rabbit	1:500	Abcam, Singapore	Ab8925
Deltex	rabbit	1:1000	Santa Cruz Biotechnology. INC	SC66858
GSK-3β	rabbit	1:1000	Santa Cruz Biotechnology. INC	SC-9166
p-GSK-3β (Ser9)	rabbit	1:1000	Cell Signalling technology	9336
p-AKT (Ser 473)	rabbit	1:1000	Cell Signalling technology	9271
NFκB/65	mouse	1:2000	Cell Signalling technology	6956
IκBα	mouse	1:1000	Cell Signalling technology	4814
iNOS	mouse	1:1000	BD Transduction laboratories	610432
TNF-α	rabbit	1:1000	Chemicon	AB1837
Cox2	rabbit	1:2000	Chemicon	AB5118
MCP-1	rabbit	1:1000	Santa Cruz Biotechnology. INC	SC-28879
IL-1β	rabbit	1:1000	Chemicon	AB1832P
Β-actin	mouse	1:10000	Sigma-Aldrich	A5316

ELISA was carried out to assess some of the cytokines secreted by BV-2 cells in the cell supernatant after different treatments together with groups stimulated with LPS only and control. For this purpose, ELISA kits for mouse TNF-α (Quantikine ELISA, R&D systems), and IL-10 (Mouse IL-10 ELISA Kit, Sigma-Aldrich) were used following the instructions in the manual provided by the respective manufacturer.

### Immunofluorescence labeling of activated Notch-1 (NICD), p-GSK-3β, NF-κB/p65 and CD11 in primary microglial cells after different treatments

To confirm that the results in BV-2 cell line was reproducible we have used the primary microglial cells in freshly prepared microglia derived from the newborn rat brain. The primary microglia would represent better the microglia in the developing brain. For this purpose, postnatal (day 3–5) rats were anaesthetized with Ketamine 75mg/kg and Xylazine 10mg/Kg cocktail and primary glia cell cultures were prepared. The brains were dissected under sterilized condition and were collected into a dish with chilled 1XPBS containing penicillin and streptomycin to wash away blood two times. The dish was kept on ice cubes. After this, the cerebral cortex was dissected out, and the adherent meninges and blood vessels on the brain surface were removed with the fine tip of a pair of sterilized forceps. The brain tissue samples derived from 3 pups were transferred into a 50 ml centrifuge tube with 10ml dissociated medium and aspirated up and down with a disposable serological pipette. The smashed tissues with the dissociated medium were transferred to a 75 cm culture flask and placed on the shaker at 37°C at 180 rpm for 25 min. The dissociated tissue mixture was transferred through 70μM of cell strainer and collected into a 50-ml centrifuge tube placed up-straight for 3 min. The upper portion of mixture was transferred into a 50-ml tube and spun down at 1000 rpm for 10 min. After this, the supernatant was discarded and the cell pellets were reseeded into cell culture chambers. The medium was changed the next day and thereafter every 3–4 days. The cells were allowed to grow in the chamber for 10–14 days until full confluency. The mild trypsin method [2] was adopted to purify the mixture microglial cells. The upper intact layer was discarded, while the bottom layer was mainly microglial cells. The chambers with containing cells were grouped as untreated and treated with LPS, DAPT+LPS and LiCl+LPS. The above treated cells and untreated cells were fixed in 4% paraformaldehyde for p-GSK-3β, NICD, NFKB/p65, and CD11b immunofluorescence double labeling.

### Statistical analysis

The statistical significance of differences between control and treatment groups was calculated using one-way analysis of variance (ANOVA). When two groups were compared, Student's *t*-test was used. Statistical significance was determined by *p<*0.05 (*) and *p<*0.01 (**). The values represent the mean ± SD at least in triplicate.

## Results

### Effects of LiCl on GSK-3β mRNA and DAPT on GSK-3β /NICD protein expression

**Fig 1:**
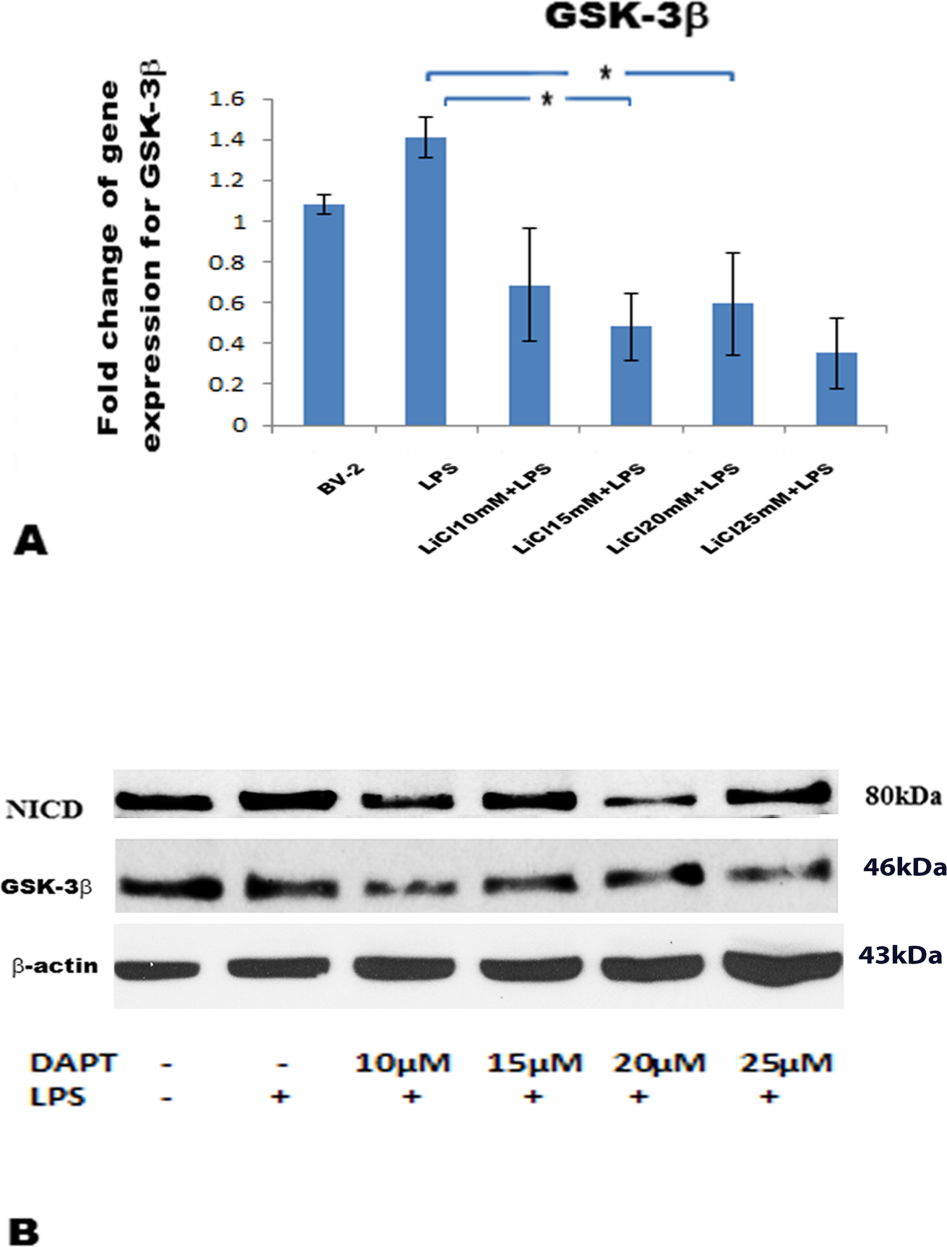
**GSK-3β mRNA (A) and NICD/ GSK-3β protein (B) expression in BV-2 microglia after pre-treatment with different doses of LiCl and DAPT, respectively, followed by LPS.** RT-PCR results show GSK-3β mRNA level was moderately increased after LPS but was suppressed significantly with LiCl at different doses compared with LPS group (A). NICD protein expression was suppressed more substantially by DAPT at 20μM; likewise, GSK-3β protein was decreased drastically by DAPT at different doses (B). The values represent the mean ± SD in triplicate.

The mRNA level for GSK-3β was moderately increased in BV-2 cells when stimulated with LPS when compared with the control cells (Fig 1A). However, GSK-3β mRNA expression was significantly suppressed in BV-2 cells when treated with LiCl at 10, 15, 20 and 25 mM followed by LPS, when compared with cells treated with LPS alone (Fig 1A). Western blot shows NICD and GSK-3β protein expression was significantly decreased in BV-2 cells treated with DAPT at 10, 15, 20 and 25μM with LPS, when compared with cells treated with the control or LPS alone (Fig 1B). The suppression of NICD expression by DAPT was most evident at 20μM compared with other treatments. LiCl at 20mM, and DAPT at 20μM were used for all subsequent experimental analysis because both dosages appeared to exert a significant effect on GSK-3β mRNA and NICD/ GSK-3β protein expression, respectively.

### LiCl decreases NICD-1, Deltex, GSK-3β protein expression along with that of proinflammatory mediators

**Fig 2:**
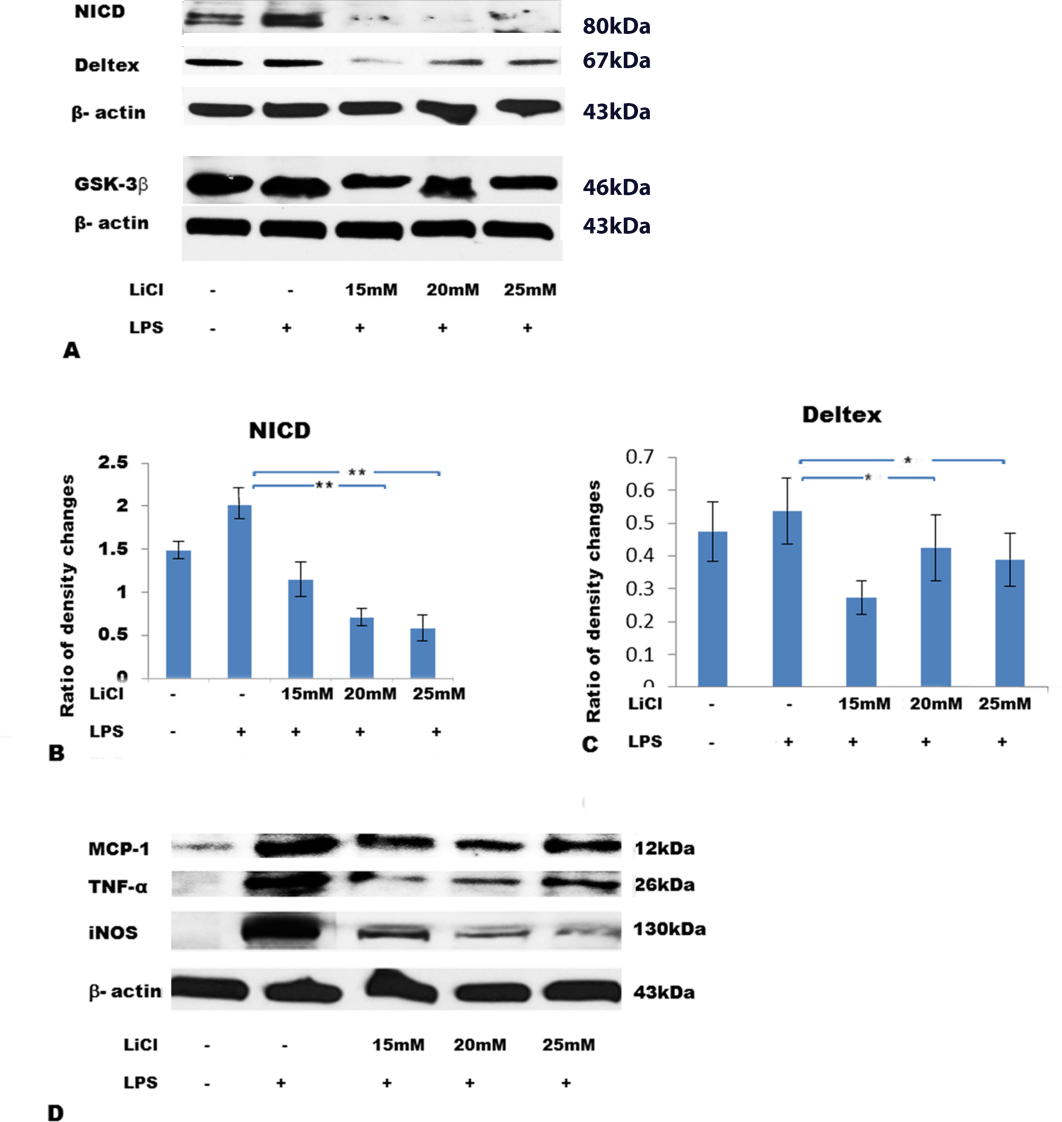
LiCl decreases protein expression levels of NICD, Deltex, GSK-3β, MCP-1, TNF-α, and iNOS in a dose dependent manner in BV-2 cells challenged with LPS. A shows specific bands of NICD (80kDa), Deltex (67kDa) and GSK-3β (46kDa). Bar graphs (B, C) show ratio of density changes by Image J; Western blotting (D) shows specific bands of MCP-1(12kDa), TNF-α (26kDa) and iNOS (130kDa). Note the decrease of these proteins by LiCl treatment. The values represent the mean ± SD in triplicate.

The protein expression changes of Notch-1(NICD), Deltex, and GSK-3β in BV-2 microglial cells treated with different doses of LiCl and stimulated with LPS were analyzed. By Western blotting, the specific protein size of NICD, Deltex and GSK-3β was 80kDa, 67kDa and 46kDa, respectively, when probed with their respective antibody. The density for the respective bands was significantly decreased in BV-2 cells treated with different doses of LiCl and challenged with LPS when compared with cells treated with LPS alone (Fig 2A–2C). Concomitantly, expression of MCP-1, TNF-α, and iNOS, with their specific size band at 12kDa, 26kDa,130kDa, respectively, in BV-2 cells showed a drastic decrease when treated with different doses of LiCl and stimulated with LPS comparing with cells treated with LPS alone (Fig 2D).

### LiCl and DAPT increase p-GSK-3β and p-AKT but decrease GSK-3β, NICD and Deltex protein expression

**Fig 3:**
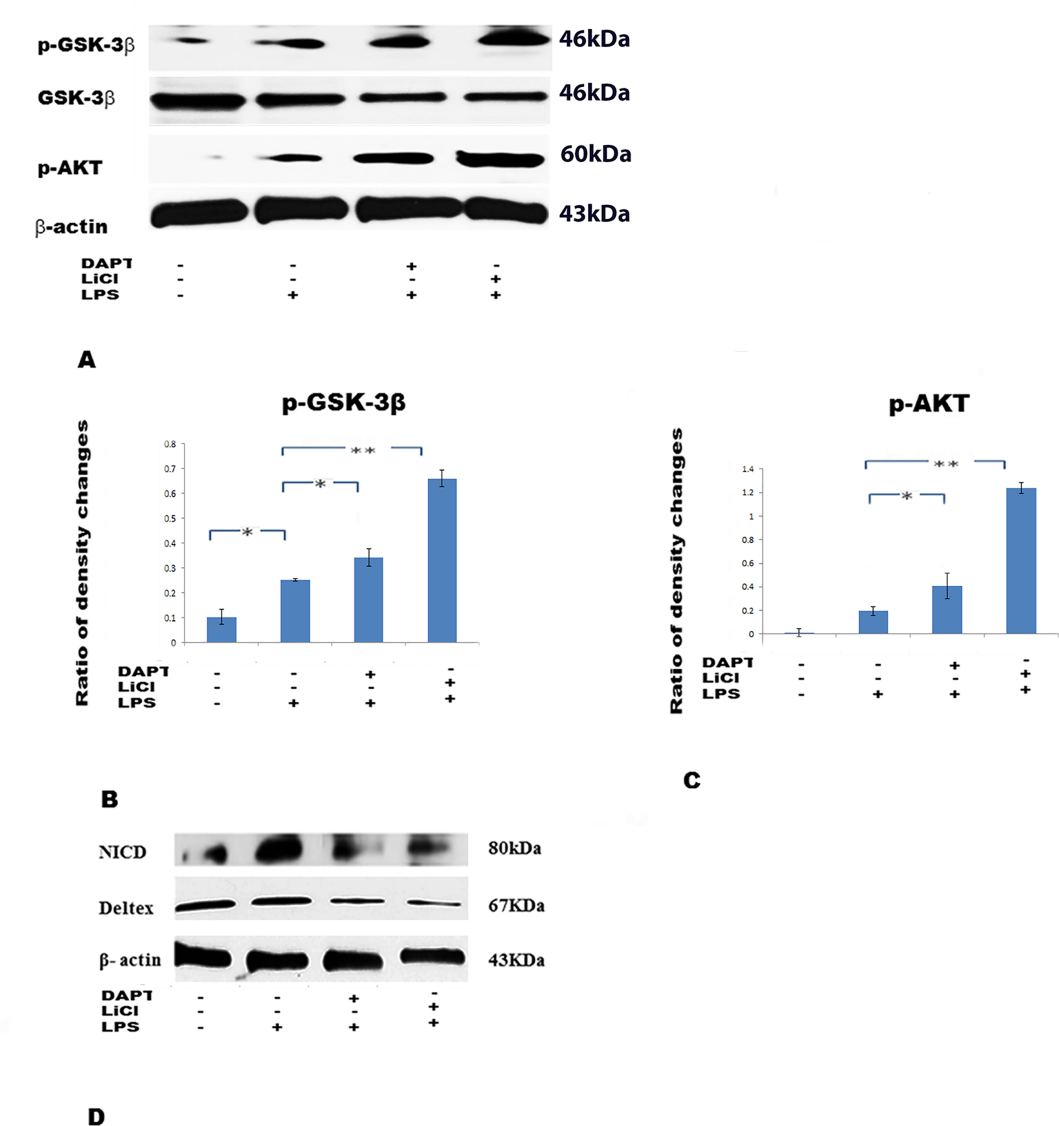
Expression changes of phosphorylated GSK-3β (p-GSK-3β)(Ser9), GSK- 3β and phosphorylated-AKT(p-AKT)(Ser 473) following treatment with LiCl (20mM) and DAPT (20μM) of BV-2 cells challenged with LPS. Western blotting (A) shows the specific bands of p-GSK-3β (46kDa), GSK-3β (46kDa) and p-AKT (60kDa). Bar graphs (B, C) show ratio of density change of specific bands for p-GSK-3β and p-AKT by Image J. (D). Western blotting shows the specific bands of NICD (80kDa) and Deltex (67kDa). The values represent the mean ± SD in triplicate.

To ascertain the interrelationship between Notch signaling and GSK-3β, DAPT (20μM) and LiCl (20mM) were used as inhibitor for Notch and GSK-3β, respectively, to pre-treat BV-2 cells followed by LPS. The protein expression for p-GSK-3β, GSK-3β, p-AKT, NICD and Deltex was then analysed. Western blotting showed p-GSK-3β, GSK-3β and p-AKT appeared at specific bands at 46kDa, 46kDa and 60kDA, respectively (Fig 3A). The band density for p-GSK-3β and p-AKT in DAPT+LPS and LiCl+LPS (Fig 3A–3C) was significantly increased when compared to LPS group; on the other hand, the band density of GSK-3β was decreased (Fig 3A). Meanwhile, the Western blotting results (Fig 3D), showed that besides reducing NICD levels (as already shown in Figs 1B and 2A), DAPT+LPS significantly reduced Deltex levels compared with LPS group. A comparable effect was observed in LiCl+LPS, as also shown in Fig 2A.

### DAPT and LiCl decrease NICD, Hes-1 and GSK-3β but enhance p-GSK-3β and p-AKT immunofluorescence in BV-2 cells

**Fig 4:**
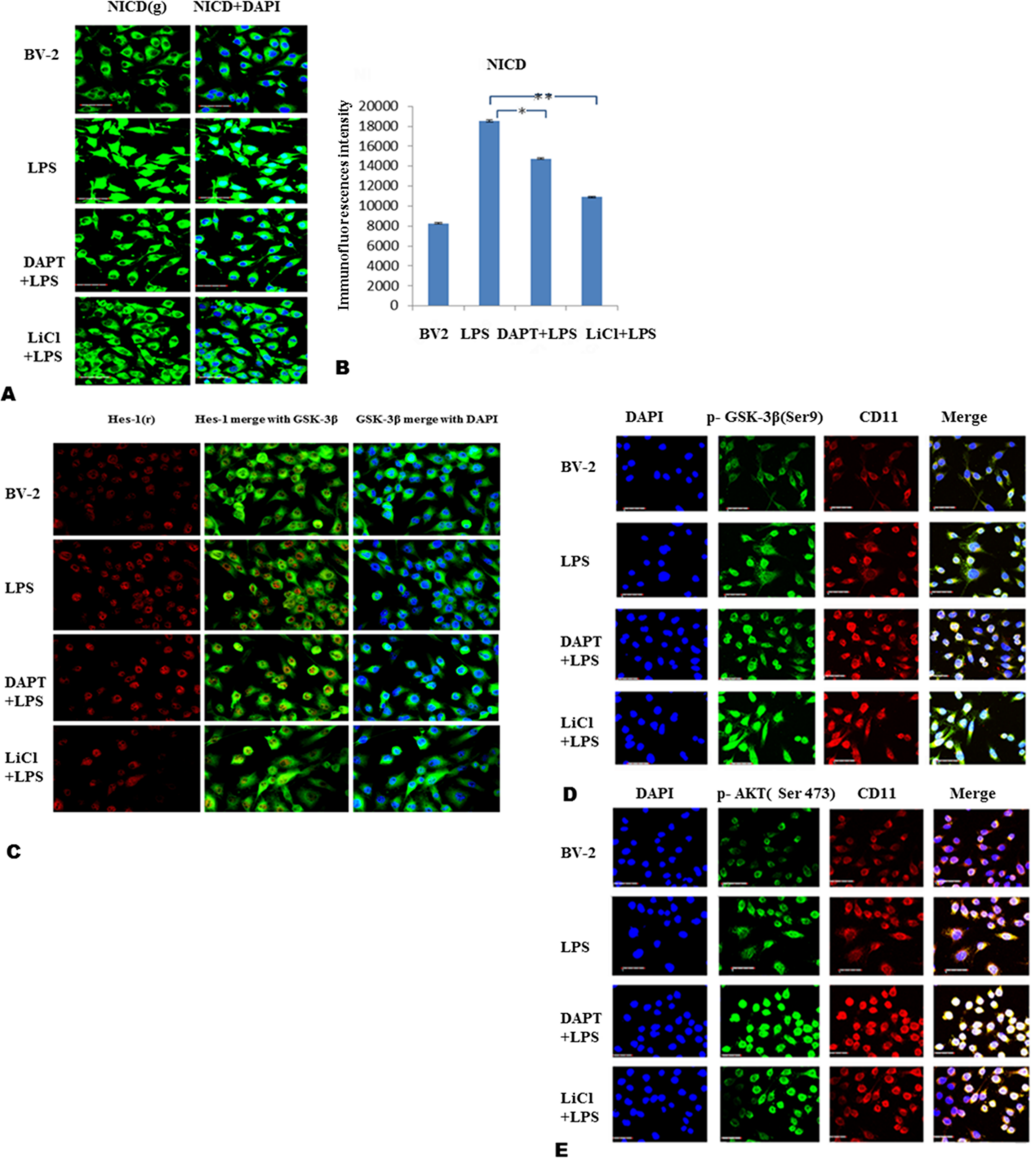
Immunofluorescence double labelling for NICD, Hes-1, p-GSK-3β (Ser9) and p-AKT (Ser473) in LPS stimulated BV-2 cells after DAPT (20μM) and LiCl (20mM) treatment. A shows immunofluorescence double labelling NICD and DAPI. Bar graphs in B show the immunofluorescence intensity of NICD by image J. C shows Hes-1 and GSK-3β immunofluorescence double labelling. Immunofluorescence double labelling for p-GSK3β/ CD11, and p-AKT/CD11 is shown in D and E. DAPI staining of nucleus is also shown. Scale bars in A, C, D and E = 40μm.

NICD immunofluorescence was markedly enhanced both in the cytoplasm and nucleus in LPS treated cells (Fig 4A and 4B); however, the fluorescence intensity in the cytoplasm, especially in the nucleus in DAPT+LPS and LiCl+LPS treated cells was noticeably attenuated (Fig 4A and 4B). In parallel to NICD, Hes-1 immunofluorescence that was augmented in LPS treated cells was decreased in DAPT+LPS and LiCl+LPS treated cells (Fig 4C). GSK-3β immunofluorescence was moderately attenuated in LPS, DAPT+LPS and LiCl+LPS compared with the control cells (Fig 4C) which is consistent with the Western blot results (Fig 3A).

Immunofluorescence signaling for p-GSK-3β and p-AKT was noticeably increased both in the cytoplasm and nucleus in DAPT +LPS and LiCl+LPS treated cells, when compared with cells treated with LPS (Fig 4D and 4E).

### DAPT and LiCl increase p-GSK-3β but decrease NICD immunofluorescence in primary microglia

**Fig 5:**
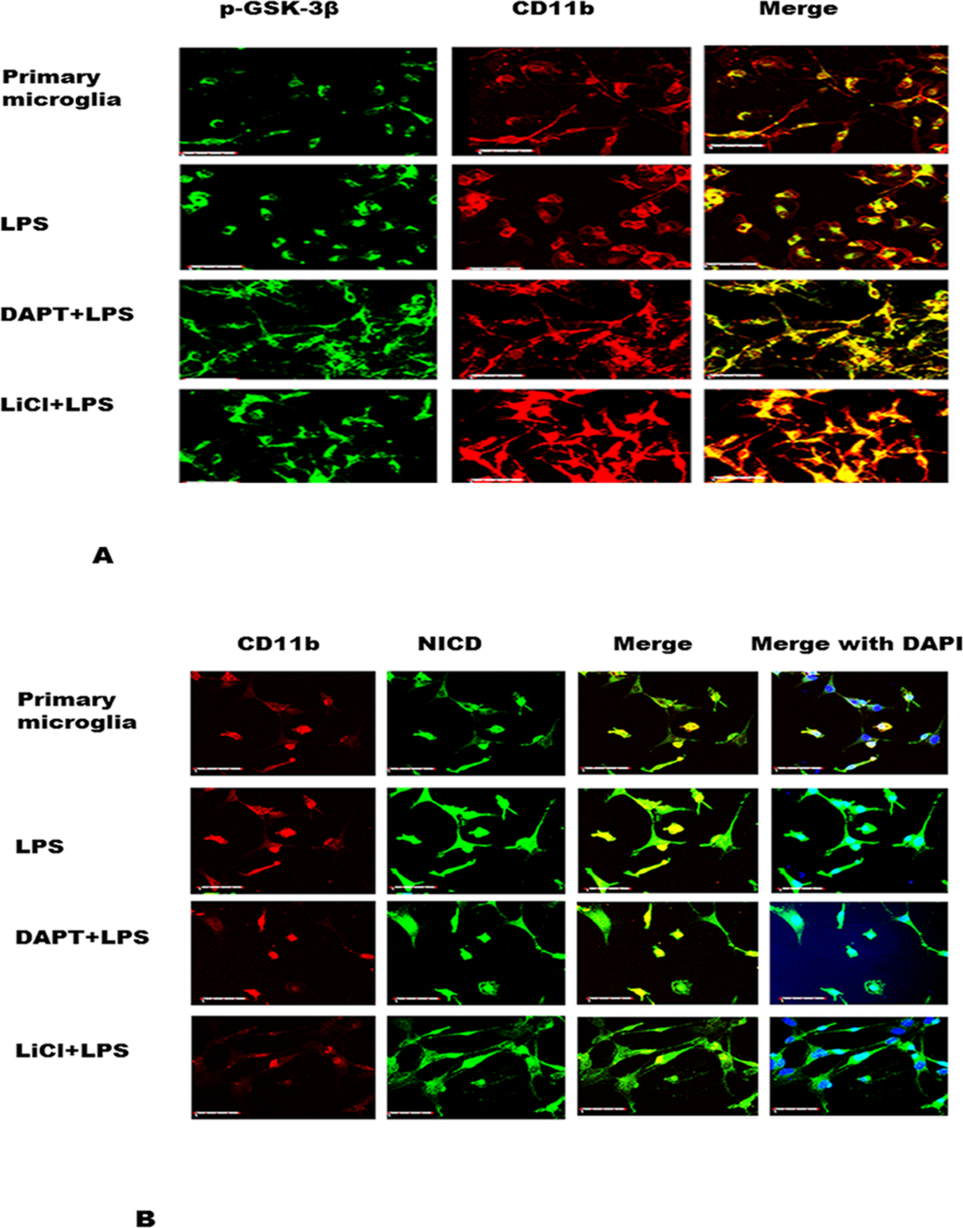
p-GSK-3β immunofluorescence is increased but that of NICD is decreased in primary microglia treated with DAPT+LPS and LiCl+LPS, respectively. A shows p-GSK- double labelling with CD11b; B shows NICD double labelled with CD11b. Scar bars in A, B = 40μm.

Immunofluorescence labeling of primary microglial cells showed that p-GSK-3β (green) and NICD (green) double labeled with CD11b (red) was noticeably increased following treatment with DAPT+LPS and LiCl+LPS in comparison with those treated with LPS alone (Fig 5A and 5B). Conversely, NICD immunofluorescence was decreased after DAPT and LiCl treatment (Fig 5B). The results confirm that in BV-2 cells (see Fig 4).

### DAPT and LiCl decrease NF-κB /p65 expression but increase that of IκBα

**Fig 6:**
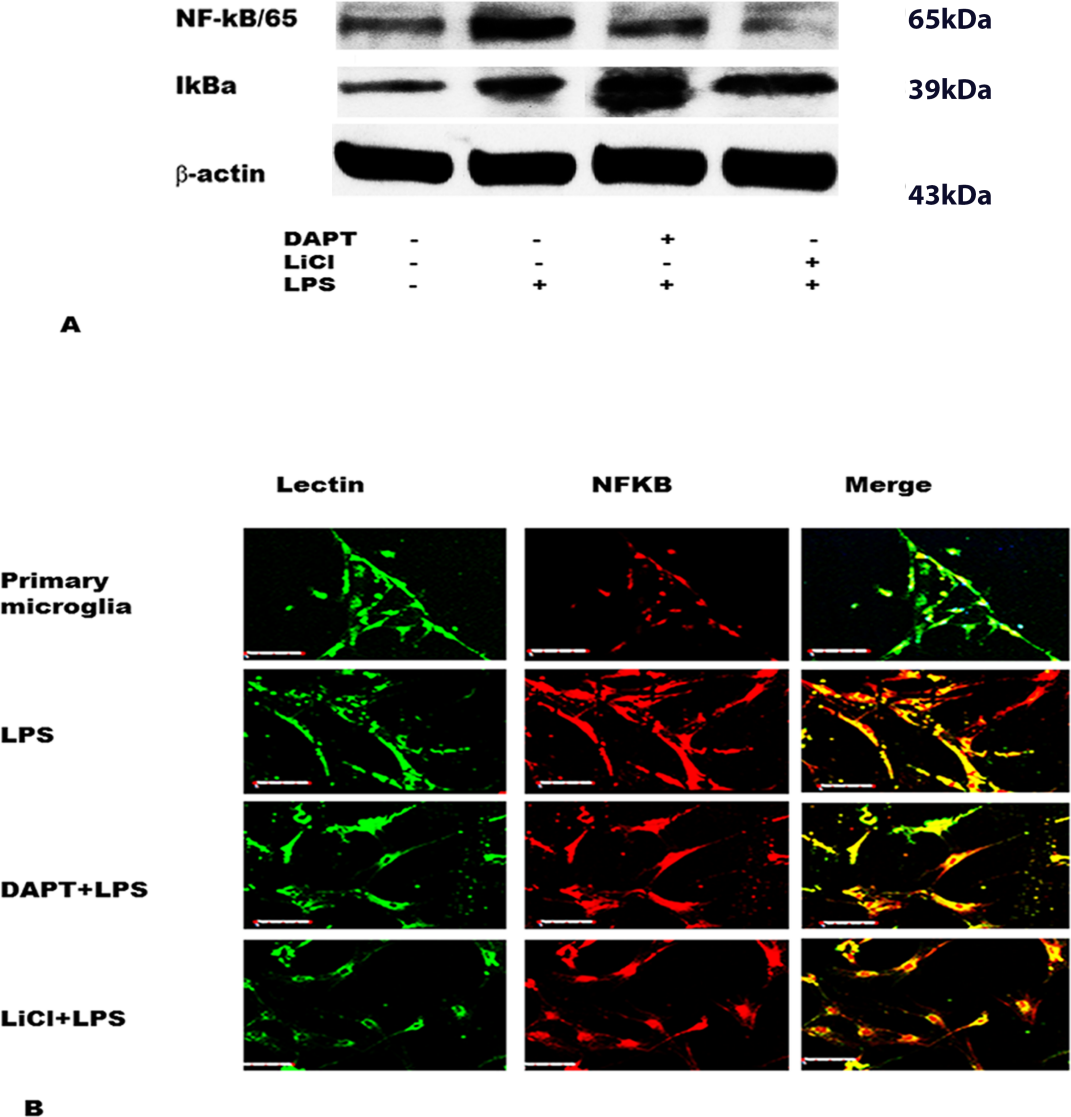
Western blotting (A) shows decrease in NF-κB /p65 but increase in IκBα expression in BV-2 cells treated with DAPT (20μM) +LPS and LiCl (20mM)+LPS. B shows NF-κB and lectin staining in primary microglia treated with LPS, DAPT+LPS and LiCl+LPS. Note the increase in NF-κB immunofluorescence by LPS which is attenuated by DAPT and LiCl. Scar bars in B = 40μm.

The protein expression level for NF-κB/p65 was increased in LPS group compared to control (Fig 6A) and decreased after DAPT+LPS and LiCl+LPS treatment comparing with LPS only (Fig 6A), while that of IκBα was upregulated in DAPT+LPS and LiCl+LPS groups compared with the LPS group (Fig 6A). Note that NF-κB/p65 immunofluorescence in primary microglia was increased after treatment with LPS; the immunofluorescence intensity was attenuated in DAPT+LPS and LiCl+LPS groups (Fig 6B).

### DAPT and LiCl decrease expression of proinflammatory mediators

**Fig 7:**
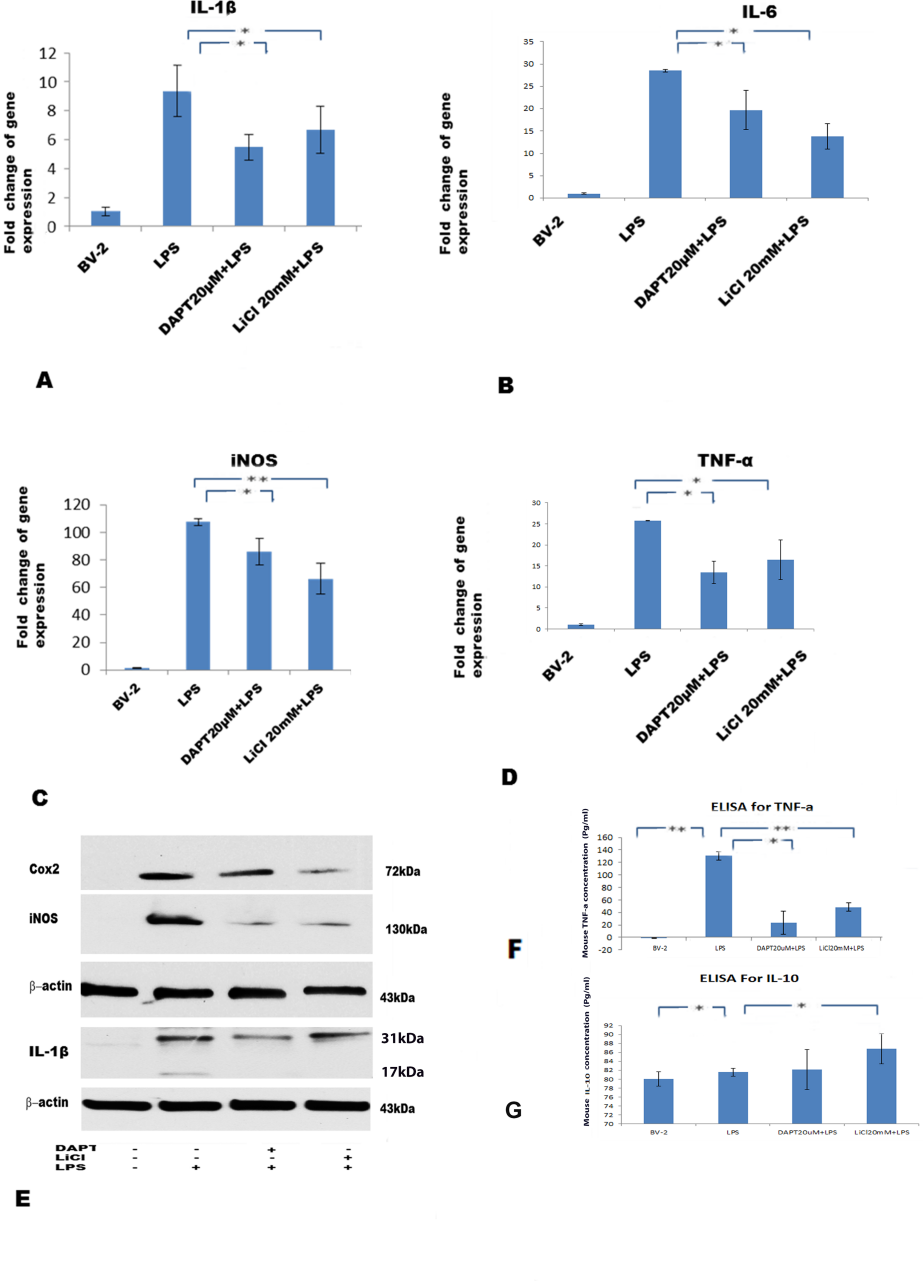
Significant reduction in gene and protein expression of proinflammatory cytokines in BV-2 cells treated with DAPT+LPS and LiCl+LPS, respectively. Bar graphs (A, B, C, D) show expression changes of IL-1β, IL-6, iNOS and TNF-α in different treatments. Western blotting (E) shows specific bands for IL-1β (31 and 17kDa), COX2 (72kDa) and iNOS (130kDa). ELISA (F) shows significant decrease in TNF-α production in the supernatant of BV-2 cells after DAPT+LPS and LiCl+LPS. ELISA (G) shows significant increase in IL-10 production after DAPT+LPS and LiCl+LPS, being more pronounced in the latter. The values represent the mean ± SD in triplicate.

By RT-PCR, the mRNA level of IL-1β, IL-6, iNOS and TNF-α was significantly decreased in BV-2 cells treated with DAPT+LPS and LiCl+LPS compared with the LPS treated group (Fig 7A and 7D). By Western blot, the protein expression level of COX2, iNOS and IL-1β was significantly decreased in the above-mentioned groups (Fig 7E). Additionally, ELISA results showed that TNF-α production was drastically reduced in BV-2 cells culture supernatant in DAPT+LPS and LiCl+LPS groups compared with the LPS treated group (Fig 7F).

### DAPT and LiCl attenuate expression of proinflammatory mediators but increase that of IL-10

**Fig 8:**
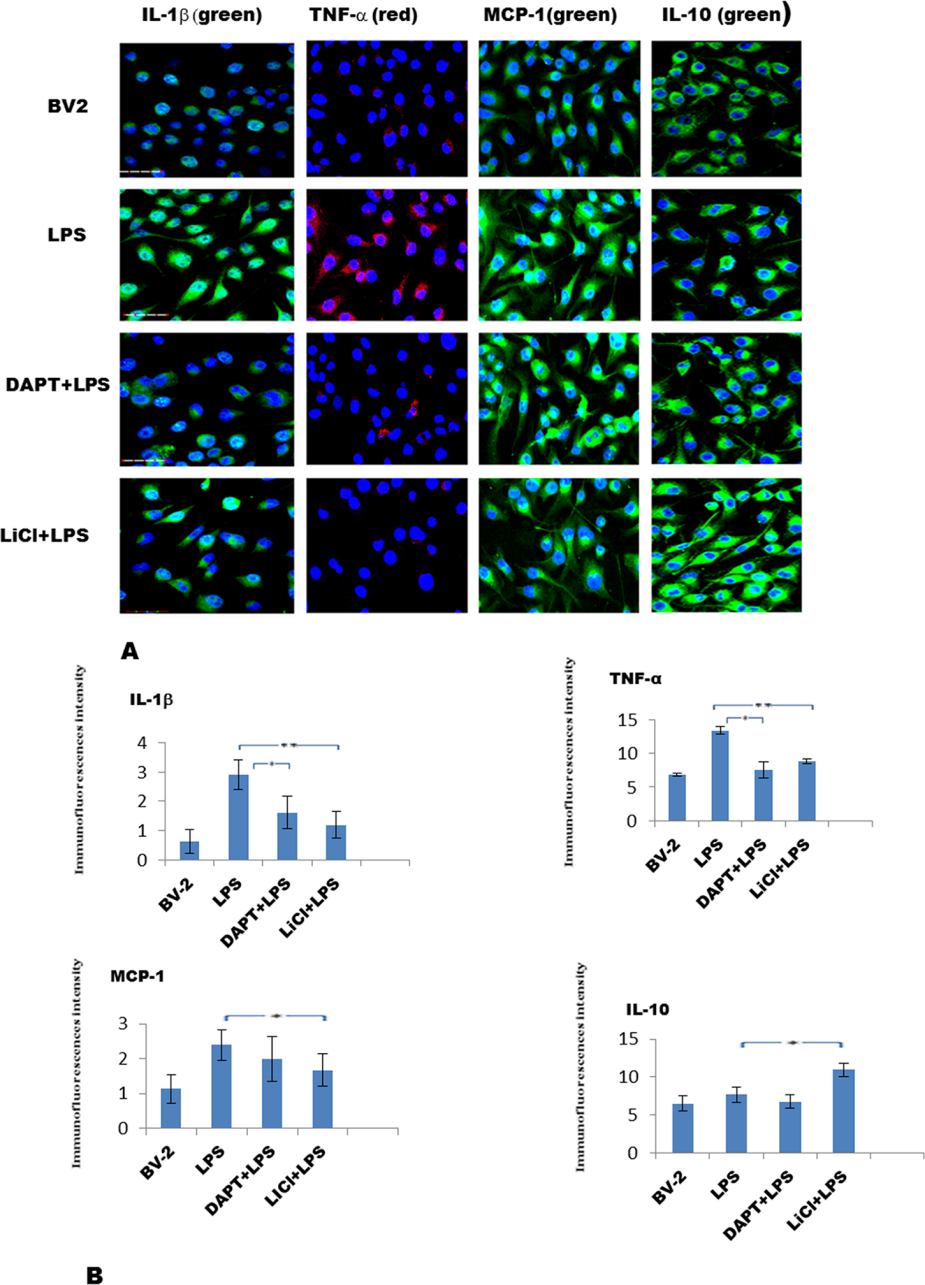
An obvious decrease in IL-1β, TNF-α and MCP-1 immunofluorescence, but increase in IL-10 immunofluorescence in BV-2 cells after treatment with DAPT+LPS and LiCl+LPS, respectively. A shows immunofluorescence for IL-1β (green), TNF-α (red), MCP-1 (green) and IL-10 (green) in BV-2 cells treated with LPS, DAPT+LPS, LiCl+LPS, respectively. Bar graphs in B show the immunofluorescence intensity changes for IL-1β, TNF-α, MCP-1 and IL-10 by Image J. Scale bars in A = 30μm. The values represent the mean ± SD in triplicate.

Double labeling showed that IL-1β (green), TNF-α (red), and MCP-1 (green) immunofluorescence intensity was noticeably decreased in DAPT+LPS and LiCl+LPS treated BV-2 cells compared with LPS treated group (Fig 8A and 8B). On the other hand, interleukin-10 expression was increased in microglia treated with LPS, DAPT+LPS, and LiCl+LPS compared with the control cells (Fig 8A and 8B). The increase was confirmed by ELISA for IL-10 being more substantial in LiCl+LPS in comparison with the LPS group (Fig 7G).

## Discussion

The present results have shown that following the inhibition of proteolysis of Notch-1 with DAPT in LPS activated BV-2 microglia, GSK-3β expression was decreased at mRNA and protein levels indicating that Notch-1 can modulate GSK-3β. Associated with this was the significant increase in phosphorylated GSK-3β (Ser9) (p-GSK-3β) in cells treated with DAPT+LPS. Very interestingly, there was also significant increase in p-AKT (ser473). Meanwhile, NF- κB /p65 protein expression was decreased, whereas that of IκBα was increased. The mRNA and protein expression levels of proinflammatory cytokines IL-1β, IL-6 and TNF-α were decreased along with that of iNOS. The results therefore support the hypothesis that Notch-1 can regulate GSK-3β and NF-κB /p65 concurrently in microglial activation in production of proinflammatory mediators.

A possible explanation for the decrease in GSK-β in microglia treated with DAPT +LPS may be due to the reduction in phosphorylated NICD caused by a decrease of NICD. Microglial cells may have to maintain an optimal level of GSK-3β for cell survival through increase in phosphorylation of GSK-3β as well as negatively feedback to promote its upstream AKT phosphorylation. This may then compensate for the decrease in AKT activity following inhibition of γ-secretase by DAPT. In this connection, it is relevant to note that Notch signalling can activate the PI3K-AKT pathway in a variety of cell lines [24].

We next explored whether GSK-3β can reciprocally regulate Notch-1 as well as NF-κB taking into consideration of the fact that the last two mentioned had earlier been reported to operate synergistically [3,7]. To ascertain this, we treated the BV-2 cells with LiCl. Remarkably, NICD and Hes-1 expression was decreased at protein level in BV-2 cells and primary microglia treated with LiCl+LPS. Furthermore, Deltex protein expression was decreased in BV-2 cells given the same treatment. Meanwhile, NF-κB/p65 protein expression level was decreased as opposed to IκBa expression that was increased in BV-2 cells and primary microglia treated with LPS+LiCl. Moreover, the mRNA expression levels of pro-inflammatory cytokines IL-1β, IL-6, TNF-α and iNOS were decreased; on the other hand, the anti-inflammatory cytokine IL-10, a marker for M2 phenotype [26] was increased. It would appear therefore that IL-10 may be differentially regulated compared with the proinflammatory mediators and that LiCl promotes polarization of microglia from M1 to M2 [26]. The present results are consistent with Foltz et al [27] who reported that GSK-3β was able to phosphorylate Notch-1 *in vitro*, and that NICD has to be phosphorylated by GSK-3β for its translocation to the nucleus [28]. In light of the above, it is suggested that Notch-1 signalling interacts with GSK-3β in regulating the production proinflammatory mediators and anti-inflammatory cytokine IL-10 in activated microglia. Meanwhile, Notch-1 is reciprocally regulated by GSK-3β as evident by the decrease in NICD and Hes-1 expression by LiCl coupled with increase in p-GSK-3β and p-AKT. This is consistent with a previous report that phosphorylation of GSK-3β down-regulates Notch-1 activity [18].

Additionally, we have shown that LiCl suppressed the expression of NF-κB/p65 and increased IκBα expression in activated microglia indicating a role of GSK-3β in modulating NF-κB /p65. We show here that DAPT and LiCl treatment had resulted in substantial increase in p-GSK-3β and p-AKT expression along with reduced production of proinflammatory mediators. It is therefore suggested that increase in p-GSK-3β and p-AKT may be related to regulating NF-κB/p65 in production of proinflammatory mediators in activated microglia, although the underlying mechanism remains to be ascertained. We have previously reported that inhibition of Notch signaling would exert its influence through TRAF6 on NF-kB/p65 [11]. However, as NF-kB activity is controlled at different levels by positive and negative regulatory elements, multiple targets may exist for the action of Notch signaling in NF-kB activity. For example, activation of phosphatidylinositol-3 (PI-3) kinase and AKT by RNS60 has been reported to rapidly upregulate IκBα via CREB that is anti-inflammatory [29]. Interestingly, we have shown that both DAPT and LiCl upregulated IκBα suggesting that suppression of NF-κB/p65 by both inhibitors may be mediated via IκBα. All in all, it is obvious that the mechanistic links between the three pathways in microglial activation are complex and remain to be fully elucidated.

In conclusion, using DAPT and LiCl as inhibitor for Notch- 1 and GSK-3β, respectively, the present results have demonstrated a complex interplay between Notch-1, GSK-3β and NF-κB/p65 signalling pathways. It is suggested that the resulting increase in p-GSK-3β, p-AKT and IκBα is involved in regulating microglia activation in production of proinflammatory mediators and/or anti-inflammatory cytokine, IL-10. It is well documented that robust microglia activation can lead to perturbation of the microenvironment especially when there is excess production of TNF-α, IL-1β, nitric oxide, glutamate etc. over a prolonged duration. This would exacerbate neuroinflammation that is implicated in different neurological diseases and disorders. Therefore, in designing of an effective therapeutic strategy aiming at amelioration of neuroinflammation mediated by activated microglia [1, 30–33], all three signalling pathways and their interrelationship among others should be addressed; indeed, targeting only one mechanism is far from sufficient.
